# Local Committees

**Published:** 1921-10

**Authors:** 


### LOCAL COMMITTEES.

An interesting conference of representatives of the voluntary hospitals in the West Riding, excluding Bradford, Leeds, and Sheffield, has been held at the Clayton Hospital, Wakefield, with reference to the appointment of two representatives upon the Local Hospital Committee for the West Riding in connection with the Voluntary Hospitals Commission. As the result of much criticism of the proposed constitution of the Local Committee, and "the extraordinary representation" given to the Commissioners and other bodies, which was held to endanger the voluntary hospitals, it was decided to form a Sub-Committee to obtain information, and to adjourn the meeting to a suitable date this month. The Chairman of the Mexborough Hospital made the remark that in his district there were no difficulties in the voluntary support, and "they were not going to hand over to the Government a flourishing concern which the people loved."

The discussion at this meeting was typical alike in attitude and in argument. At the time of writing the adjourned meeting has not been held, but we may say at once that further inquiry should modify the suspicious attitude of the West Riding. In other pages of this journal similar meetings and similar suspicions have been chronicled, and it is unnecessary in each case to repeat the reasons which in our view make these suspicions ill-founded. In themselves, however, they are a healthy sign of faith in the voluntary system, and a sign, too, which will, we think, be extremely welcome to the Ministry of Health and the Government. If the Hospitals Commission means anything at all, it means that the Government is determined not to saddle itself with the hospital question. If the inadequate Government grant means anything at all, it means that the Government is not going to subsidise the voluntary hospitals. It was given to stave off any such demand, and reduced, probably, to make that intention unmistakable.

We have previously explained why the hospitals are poorly represented upon the Local Committees. The reason is, as competent hospital witnesses explained before the Cave Committee, that hospitals by themselves have hitherto failed to co-operate together, and therefore some body familiar, but not identical, with their needs was desirable to "bring them into line." Hitherto they have thought and acted individually, and in some competition with each other. The need now is to make the best of their resources by, as it were, pooling their ideas, plans, and activities within each area. For this reason inter-hospital bodies, known as Local Committees for each area, have been devised to supply a common mind and a common policy in each district. These bodies are mainly advisory. They have neither the power nor the resources to control or "take over" the separate institutions which they exist to co-ordinate. But that their advice may not be neglected they are furnished with the power to recommend grants to the Hospitals Commission. The question really is whether this is sufficient to get their suggestions attended to. An impartial observer who considers the whole voluntary system may rather fear that they are too weak and not that they are too strong for their task. At present there seems little reason to dread their interference.

### THE GENERAL NURSING COUNCIL.

The coming winter opens up a vista of considerable interest for the nursing profession. It will see the firstfruits of the training in general nursing under the syllabus of the General Nursing Council, and the issue of the syllabuses of training for the nursing of sick children, for fever nurses and for male and mental nurses; those for sick children and fever have indeed been approved by the Council, and are now ready, Dr. Goodall and Miss Villiers having been officially thanked for the able syllabus for fever nurses which they drafted. The Education Committee of the Council is taking the expert advice of Miss Beadsmore Smith, Matron-in-Chief at the War Office, in drafting the syllabus of training for male nurses, and the Mental Nursing Committee likewise desires advice before framing its regulations for the admission of future nurses to the supplementary part of the Register for mental nurses; the lack on the Council of a matron or female head nurse of a mental hospital is a handicap in drafting these regulations, and it has been decided to obtain necessary expert advice.

The General Nursing Council and its committees have plenty of work in front of them; the State Register is now open, and up to September 30 366 nurses had been placed on the General Part, fourteen on the Fever Nurses' Register, and one on the Mental Nurses' Register; in all 1,816 applications had been received up to that date. The Rules for Registration can be purchased from the Registrars of the respective Councils, and they should be carefully studied. It is interesting in this connection to note that the General Nursing Council has power to request any of its members to visit any place for the purpose of explaining the Act or the Rules. Matrons who desire such a visit would therefore do well to communicate with the Registrar at 12 York Gate. Manchester and Liverpool have already requested such a visit, which is being made by Mrs. Bedford Fenwick as we go to press. It is much to be hoped that within the next few months a plan for grouped training and the affiliation of small and special with large hospitals and infirmaries will be evolved and agreed; considerable anxiety is felt by those responsible for the nursing in the former institutions which it were well to allay as soon as possible. The General Nursing Council also has in hand the issue of a list of approved training-schools, and of an examination syllabus.

---

The Secretary of State for Scotland has decided that the rules drafted by the Scottish Council must stand and that nurses trained in fever hospitals are not eligible for admission to the General Register.

